# Supplementary material for: The interactome of intact mitochondria by cross-linking mass spectrometry provides evidence for coexisting respiratory supercomplexes
Source: Mol Cell Proteomics. 2017 Dec 8;17(2):216–32. doi: 10.1074/mcp.RA117.000470 (PMC5795388; doi:10.1074/mcp.RA117.000470)
Supplement: Supplemental Data [file supp_RA117.000470_133922_0_supp_23978_fzffwf.zip › spectra_annotation/mito_spectra_annotation/318-1-4-1-2-1.pdf]

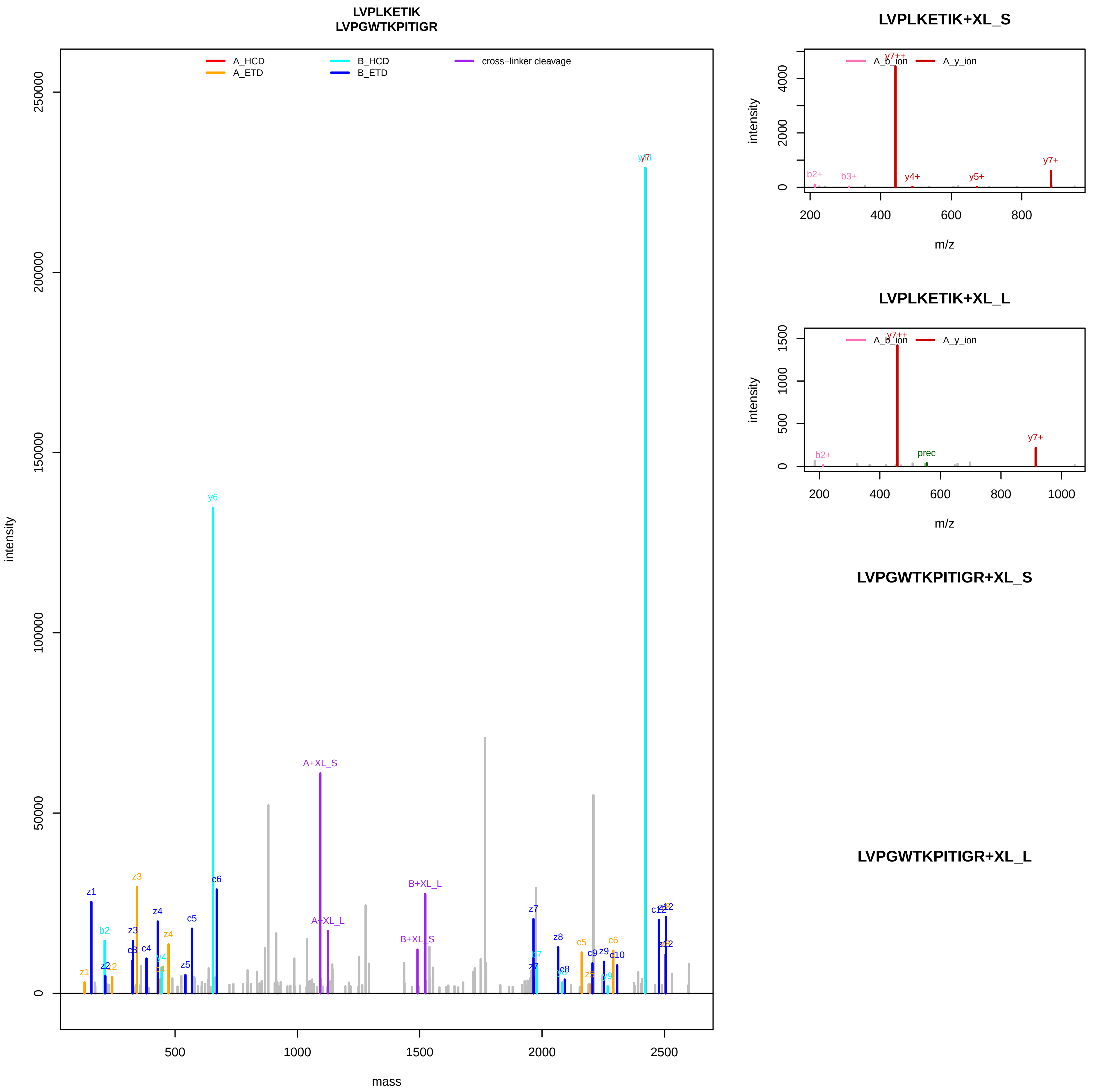

LVPLKETIK
LVPGWTKPITIGR
A_HCD
A_ETD
B_HCD
B_ETD
cross−linker cleavage
intensity
mass
0
50000
100000
150000
200000
250000
500
1000
1500
2000
2500
y6
y7
A+XL_S
A+XL_L
B+XL_S
B+XL_L
z1
b2
z2
z3
c3
c4
z4
y4
c4
z5
c5
c6
z7
y7
z8
c8
c5
z5
c9
z9
y9
c6
c10
c12
z12
LVPLKETIK+XL_S
A_b_ion
A_y_ion
intensity
m/z
0
2000
4000
200
400
600
800
b2+
b3+
y7++
y4+
y5+
y7+
LVPLKETIK+XL_L
A_b_ion
A_y_ion
intensity
m/z
0
500
1000
1500
200
400
600
800
1000
b2+
y7++
prec
y7+
LVPGWTKPITIGR+XL_S
LVPGWTKPITIGR+XL_L
